# Lean Body Mass Harbors Sensing Mechanisms that Allow Safeguarding of Methionine Homeostasis

**DOI:** 10.3390/nu9091035

**Published:** 2017-09-20

**Authors:** Yves Ingenbleek

**Affiliations:** Laboratory of Nutrition, Faculty of Pharmacy, University Louis Pasteur, F-67401 Strasbourg, France; ingen@unistra.fr; Tel.: 0033-467-74-87-17

**Keywords:** lean body mass, transthyretin, protein malnutrition, inflammatory disorders, methionine pool size, cystathionine-β-synthase, homocysteine, sarcopenia

## Abstract

Protein-depleted states generate allosteric inhibition of liver cystathionine β-synthase (CBS), which governs the first enzymatic step of the transsulfuration cascade, resulting in upstream accretion of homocysteine (Hcy) in body fluids. A similar Hcy increase may arise from normal hepatocytes undergoing experimentally-induced impairment of betaine-homocysteine methyltransferase (BHTM) activity or from components of lean body mass (LBM) submitted to any inflammatory disorder. LBM comprises a composite agglomeration of extrarenal tissues characterized by naturally occurring BHTM inactivity. As a result of cellular injury, LBM releases high concentrations of Hcy into the extracellular space, contrasting with the disruption of normal remethylation pathways. Hyperhomocysteinemia acts as a biomarker, reflecting the severity of insult and operating as an alarm signal. Elevated Hcy levels constitute a precursor pool recognized by a CBS coding region that reacts to meet increased methionine requirements in LBM tissues, using its enhanced production in hepatocytes. Preservation of methionine homeostasis benefits from its high metabolic priority and survival value.

## 1. Introduction

Human body composition studies indicate that nitrogen (N) is a key component of all proteins found in body tissues [1]. Protein synthesis requires the sequential arrangement of amino acid (AA) building blocks via translation processes, as dictated by the genetic code. Twenty canonical AAs have been identified in human proteins, of which eight are indispensable amino acids (IAAs), implying their obligatory supply in customary diets. Methionine (Met) and cysteine (Cys) represent the 2 *S*-containing AAs (SAAs) provided by dietary items. Cys can furnish part of the total SAA needs of mammals [2], thereby ensuring a sparing effect of at least half the dietary Met requirements [3]. The usually recommended dietary allowances for both SAAs are 13 to 16 mg kg^−1^ day^−1^ or approximately 17 to 27 mg/g protein. Thus, the proportion of Met in mixed body proteins reaches a mean of 2%–3%. We have collected data showing that, in contrast to the seven other IAAs, Met benefits from unique homeostatic mechanisms with survival value under conditions of chronic protein deprivation or excessive N body losses [4].

## 2. Main Methionine Characteristics

**Figure 1:**
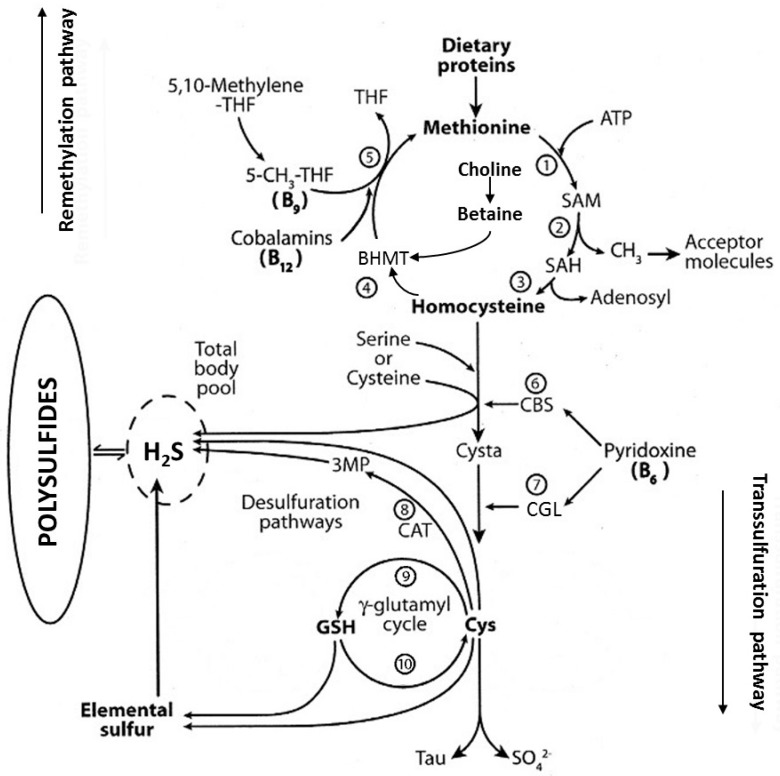
Main endogenous pathways of methionine metabolism. **Compounds:** ATP, adenosyl-triphosphate; Cys, cysteine; Cysta, cystathionine; GSH, glutathione; H_2_S, hydrogen sulfide; 3MP, 3-mercaptopyruvate; SAH, *S*-adenosyl-homocysteine; SAM, *S*-adenosyl-methionine; Tau, taurine; THF, tetrahydrofolate; SO_4_^2-^, sulfate oxyanions. **Enzymes:** (1) Met adenosyl-transferase; (2) CompoundsSAM methyltransferase; (3) adenosyl-homocysteinase; (4) BHMT, betaine-homocysteine-methyltransferase; (5) MS; methionine-synthase; (6) CBS, cystathionine β-synthase; (7) CGL, cystathionine γ-lyase; (8) γ-glutamyl-synthase; (9) γ-glutamyl-transpeptidase (adapted from [4]).

Met has a molecular mass (MM) of 149.22 g/mol and is the sole IAA that provides S in dietary products that are currently consumed by humans. The mean protein concentration of plant products is 9.8% or 1.56 g N% using a conversion coefficient of 6.25. The mean Met content in plant products is 1.27% per g protein [5]. The mean protein concentration of animal products is 16.5% or 2.64 g N%. The mean Met content in animal products is 3.17% per g protein, defining S:N molar ratios ranging from 1:13 to 1:18 [5]. On a weight basis, the dietary intake of N and Met by plant proteins is approximately half that of animal products, displaying S:N molar ratios situated between 1:20 and 1:35 [5] and showing that vegetables do not optimally fulfill human tissue requirements. The dietary gap is documented by chronic shortage in Met intake observed in vegans [6], leading to the concept of unachieved replenishment of lean body mass (LBM) tissues [7]. Under well-balanced dietary regimens, the proportion of ingested Met incorporated into the biosynthesis of body proteins represents 80–90% of intake. The remaining Met fraction may undergo three distinct metabolic pathways. The first is *transmethylation* (TM), a biological pathway of great qualitative importance whose activity depends on the release by Met of bioavailable methyl (CH_3_) groups [8] (Figure 1). The TM pathway requires the condensation of Met to ATP to yield the dephosphorylated *S*-adenosyl-methionine (SAM) molecule, a primary biological CH_3_ donor that is involved in over 100 TM acceptor substrates. SAM molecules may in turn undergo demethylation to release *S*-adenosyl-homocysteine (SAH), the enzymatic hydrolysis that permits recovery of the SAM moiety and the production of homocysteine (Hcy), a nonproteinogenic AA that occupies a branch-point from where both *transsulfuration* (TS) and *remethylation* (RM) pathways originate. Under physiological circumstances, healthy adults who consume well**-**balanced diets with appropriate Met intake levels undergo splitting of TS and RM processes into nearly equivalent proportions [9,10] regulated by SAM assuming switch functions between competing pathways [11]. In response to high Met intake, the SAM concentration increases in liver cells, causing allosteric overstimulation of cystathionine-β-synthase (CBS, EC 4.2.1.22) and irreversible overflow of Hcy molecules along the TS cascade [12]. In that context, the activity of both enzymes involved in Hcy→Met remethylating processes, namely betaine-Hcy-methyltransferase (BHMT, EC 2.1.1.5) and methionine-synthase (MS, EC 2.1.1.13), are concomitantly downregulated [12].

The intracellular concentration of SAM also inhibits the activity of methylene-tetrahydrofolate reductase (MTHFR, EC 1.7.99.5) which converts the precursor [N_5_,N_10_]-MTHF molecule 5-methyl-tetrahydrofolate (MTHF, folates) [13]. This last compound releases CH_3_ groups allowing for the methylating activity of MS to be stimulated, which is ubiquitously present in all cells and working in concert with methylcobalamin (vitamin B_12_) acting as a prosthetic group. BHMT occurs only in the liver of all species and the kidneys of primates and pigs [14] which use CH_3_ groups released by the dietary intake of betaine or from choline oxidation. CBS, which governs the first step of the TS pathway, may condense Hcy to serine, forming cystathionine which in turn is converted by cystathionine-γ-lyase (CGL, EC 4.4.1.1.) to Cys and hydrogen sulfide (H_2_S) [15] with pyridoxal-5′-phosphate (PLP, vitamin B_6_) working as a co-factor for both CBS and CGL enzyme activities (Figure 1). A third desulfuration pathway generating H_2_S from Cys is also described following an intermediate 3-mercaptopyruvate (3-MP) converting step [16]. In addition, elemental sulfur (S) found in tap waters may be subjected to non-enzymatic Cys and GSH reducing activities, allowing nascent H_2_S delivery [17]. All H_2_S sources flow into a barely detectable free body pool that is in reversible equilibrium with intracellular polysulfide (H_2_S_n_) compounds that serve as H_2_S storage sites [4,18]. The Cys-sulfinate oxidative pathway finally releases hypotaurine, taurine, and SO_4_^2−^ oxyanions as TS end-products [19].

## 3. Methionine in Protein-Depleted States

Transthyretin (TTR) is a valuable biomarker of protein-depleted states [20]. Its small pool size (10 mg/kg body weight), which is mainly confined to the intravascular space, and its short half-life of 2 days [21] explains its early response to any alteration in protein status. The serum analyte is now widely used in developed countries to detect hospitalized patients who require dietary management [20]. The TTR indicator also allows for the monitoring of all forms of protein-energy malnutrition, from emaciated marasmus to edematous kwashiorkor [20]. A field study undertaken in Western Africa compared the respective clinical usefulness of TTR [22] and free plasma IAAs advocated as indices of protein status [23]. The site of investigation was a goitrous area characterized by evenly distributed iodine-deficiency which therefore is not accountable for differences in thyroid swelling. Using World Health Organization criteria, 105 adult vegetarian subjects were recruited and stratified into stages I, II, and III of goitrous hyperplasia. Results of the nutritional survey were the first to demonstrate that Met plasma values remained stable within the normal range at the expense of a gradual Hcy elevation, in contrast with the stepwise downregulation of the seven other IAAs [22]. The data contend the view that chronically deficient N and Met intakes may contribute to the worsening of the goitrous processes following biological mechanisms elsewhere described [24]. A second comparable field study was performed in Central Africa, focusing more specifically on the three hydrosoluble B vitamins that regulate the TS and RM pathways [25]. Here, too, methioninemia was maintained within normal limits, whereas Hcy underwent a sequential increase negatively correlated with the drop in TTR plasma concentrations [26]. Folate and pyridoxine levels of these patients were satisfactory, whereas cobalamin concentrations were kept at the lower threshold of normal and regarded as unlikely to be responsible for the elevated Hcy concentrations [25]. These findings do not match the overall consensus that vitamin B_12_-deficiency is a major scourge in vegetarian populations worldwide [27], mainly in Asian countries [28,29,30] but also in Western vegan subjects [31,32] who have adopted, for socio-cultural or political motivations, the choice of restricting their diets to plant products. Indian workers underwent therapeutic trials that compared pharmacological doses of oral B_12_ over a short time (500 µg every alternate day/6 weeks) [29] to physiological doses in long-term investigations (2 or 10 µg day/year) [30]. Both assays provided evidence that B_12_ fortification was equally efficient in reducing, but not normalizing plasma Hcy concentrations. We assume that the persistence of elevated Hcy values in cobalamin-replete subjects manifesting unresponsiveness to additional B_12_ intake [29] constitutes a valuable indication of LBM depletion [25], which may be assessed by the serial measurement of lowered TTR levels [26].

Both African surveys [22,26] show that the preservation of Met homeostasis in protein-depleted states is promoted by the CBS-induced upstream accumulation of Hcy in biological fluids, which serves as a precursor pool that can be readily driven into RM processes [25]. SAM-induced allosteric alterations of the Michaelis constant affinity of CBS, BHMT, and MS for Hcy cause synergistic reorchestration of TS and RM enzyme activities [33]. Two recent experiments using mouse [34] and rat [35] strains submitted to Met-restricted regimens have confirmed that the TS pathway is impaired at the end of the deprivation period with an upstream elevation of Hcy and substantial body weight (BW) loss of 38% and 44%, respectively. These data are reminiscent of balance studies performed on pig models, which showed that withdrawal of Met and Cys from otherwise normal diets causes the greatest LBM depletion, nearly equal to that generated by protein-free diets [36].

## 4. Methionine in Excessive LBM Losses

**Figure 2:**
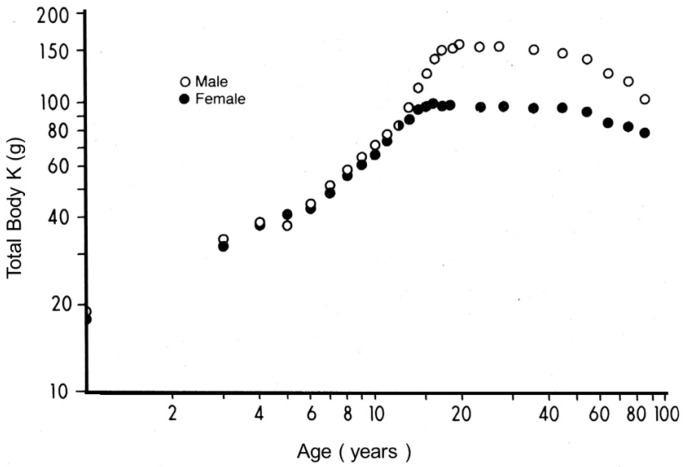
Evolutionary patterns of lean body mass (LBM) values throughout the human lifespan. Compilation of seven different clinical investigations performed in healthy subjects from birth to very old age and showing body accretion of total body potassium (TBK) values determined by the measurement of the naturally occurring radioisotope ^40^K using dual-energy X-ray absorptiometry (DXA). The results are plotted against age on double-logarithmic coordinates. Ninety-five percent (95%) of TBK is sequestered within metabolically active tissues and narrowly correlated with total body N (TBN), making this last parameter a valuable tool to appraise LBM values in health and disease (Forbes, [1]). Figure 2 shows that normal TBK concentrations are approximately 140–160 g in adult men and 90–110 g in adult women, yielding male/female K and N ratios of approximately 1.4:1.

**Figure 3:**
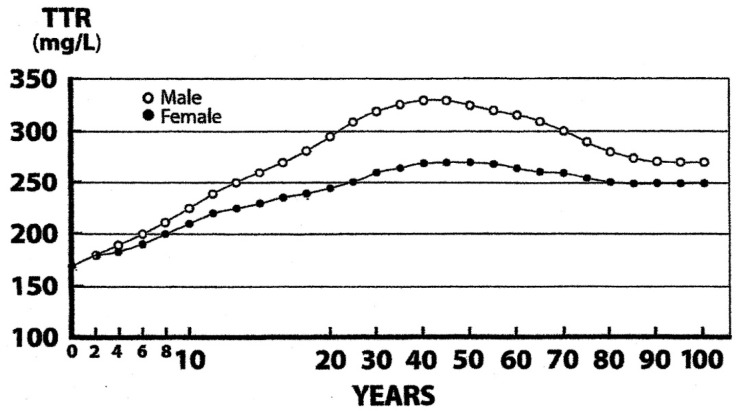
Evolutionary profiles of transthyretin (TTR) concentrations throughout the human lifespan. Evolutionary profiles of TTR concentrations measured in the blood samples from 67,720 healthy U.S. citizens using immunoturbidimetric analysis (Bienvenu et al., [40]). TTR and LBM values manifest closely superimposable trajectories. The figure shows the lowest values measured at birth, linear progression without sexual difference until the onset of puberty, occurrence of sexual dimorphism with more pronounced rise in adult males because of a larger musculature, followed by plateau levels until the age of 60 years, and finally gradual downsizing toward sarcopenia in both sexes, with a steeper slope observed in elderly males. Both TTR and LBM curves show comparable abrupt S-shape elevations from the onset of adolescence until the beginning of adulthood, which are partially obliterated due to changes in the graduation of the abscissa scales.

The most convenient approach to human body composition is a binary system consisting of fat mass (FM) and LBM obtained by subtracting FM from BW. Dual-energy X-ray absorptiometry (DXA) technology is the gold standard to assess human body composition [1] and is based on the measurement of β-rays emitted by the naturally occurring radioisotope **^40^**K, 95% of which is confined within metabolically active tissues. DXA provides an accurate assessment of LBM status, assuming that the measured total body potassium (TBK) values are narrowly correlated to those of total body nitrogen (TBN) within an average K/N ratio of approximately 3 mEq K/g N [37], indicating that TBK is a valuable tool to define TBN in health and disease (Figure 2). In a healthy reference man weighing 70 kg, LBM contains the bulk of TBN (1800 g or 64 mol) and TBK (140 g or 3600 mmol) [38]. LBM appears as a composite agglomeration of organs and tissues schematically subdivided into a *visceral* protein compartment, with the liver, the small intestinal mucosa, and thymoleukocytic tissues as main components characterized by rapid turnover rates and synthetic capacities; and a *structural* protein compartment comprising muscle mass, skin, and connective or cartilaginous appendages distinguished by slower turnover rates [39]. The measurement of plasma TTR from birth to death in a large group of healthy US citizens reveals strikingly similar age- and gender-related profiles superimposable to TBK correlates (Figure 3) [40], indicating that TTR data operate as surrogate indicators of LBM values in health and disease [7]. The recent proposal to select TTR as a substitute for LBM [7] is supported by several clinical investigations, notably conducted in kidney patients, revealing that LBM [41] and TTR [42] are equally informative in grading the progression of morbid processes and predicting lethal outcomes.

Adult human studies have shown that the liver has an oxygen consumption rate (44 mL 0_2_/kg) approximately 20-fold higher than muscle mass (2.3 mL 0_2_/kg) [43], pointing to a much faster protein turnover rate in the former organ. However, when comparing the size of the liver (2.6% of BW or 1.7 kg) with that of muscle mass (37% of BW or 26 kg), both organs contribute evenly to the daily basal metabolic activities, estimated at 26.4% and 25.6%, respectively [43]. These pioneer studies were recently confirmed in a clinical investigation that showed that the two predominant organs of both visceral and structural compartments are key determinants of resting energy expenditure (REE), working together to generate 50% of the total body basal metabolic cost [44]. There exists an overall agreement that fluctuations in REE values are mainly attributed to alterations in size and composition of metabolically active LBM components.

Inflammatory disorders of any cause are initiated by activated leukocytes that release cytokines as autocrine, paracrine, and endocrine molecules [45]. Proinflammatory cytokines regulate the overproduction of acute-phase reactants (APRs), which contribute to defense and repair mechanisms with specific kinetic and functional properties [46]. During the course of any inflammatory disorder, the overall protein metabolism of the stressed body is altered, and the turnover of most proteins at the site of injury is accelerated leading to an overabundance of several N-catabolites in the urinary output. As a result, excessive LBM losses gradually develop best identified by the serial measurement of TTR [7]. Interleukin-6 indeed significantly suppresses TTR synthesis, as documented in animal [47] and clinical [48] experiments. Major stressful disorders are associated with massive urinary excretion of elemental sulfur [49], depleting total body (TBS) reserves estimated at 4400 mmol (140 g) in the reference male [38] while maintaining tightly correlated ratios with TBN in human tissues [4,5]. The measurement of sulfur and N urinary output in trauma patients indeed yields values close to the 1:14 ratio [50] that characterizes mammalian tissues [5], indicating that TBN and TBS stores undergo concomitant breakdown patterns throughout the course of injury. In such catabolic states, downregulation of the Met pool sizes likely remains narrowly correlated with the diminished TBN reserves. Metabolic investigations using stable isotopic material have shown that severely burned patients undergo strong overstimulation of the three TM, TS, and RM pathways [51]. The septic burden imposed on animal models similarly causes marked acceleration of all functional steps depending on the TS cascade—notably those involving Cys and GSH turnover rates [52,53]—synthesis of H_2_S, and clearance of reactive oxygen species (ROS) by-products. Despite great metabolic upheaval affecting septic or injured patients [4], unaltered methioninemia is maintained, whereas most other plasma AA values are reduced by 10% to 30% [54]. These data confirm the stringent regulatory mechanisms that control Met homeostasis.

## 5. Salvage Mechanisms for Methionine Homeostasis

Hyperhomocysteinemia (HHcy) associated with inflammatory burden of any cause is a currently described clinical condition found in hospital settings. One of the first observations referred to critically ill patients who were undergoing therapeutic management in intensive care units and displaying significantly increased plasma Hcy values [55]. The patient chart encompasses all ages, genders, and inflammatory factors that may cause undifferentiated chronic, subacute, or acute disorders. In cancer patients, the elevation of Hcy values was reportedly regarded as a reflection of disease progression, culminating when its course is maxed out while subsiding after successful recovery [56]. This last clinical description suggests a direct negative correlation between LBM size and HHcy states, regardless of folate status. Comparable conclusions can be drawn from any clinical condition that is characterized by regressive changes of body organs as a result of normal aging or degenerative processes. A good example is the progressive downsizing of muscle mass [57], which represents the main LBM structural component [43]. Stepwise sarcopenia in elderly persons during aging is predicted to be genetically programmed [58] and accompanied by lowered TTR plasma values [40] diverging with increasing Hcy concentrations [59]. Apparently healthy elderly subjects show a wide distribution range of Hcy values, with some exceeding 35 µmol/L [59]. This period of life is characterized by the presence of low grade circulating levels of cytokines and APRs [60], suggesting that a rampant inflammatory burden may further deteriorate the sarcopenia of elderly persons. Moreover, the elevation of Hcy levels is significantly accelerated in old subjects who suffer overt inflammation [61]. These data indicate cytokine-induced urinary spillover of N-catabolites, causing additional depletion of LBM stores and muscle mass. It is here tempting to speculate that the shrinking of the intramuscular Met pool size, resulting from progressive sarcopenia, may reflect a state of subclinical malnutrition remaining undetected. That working hypothesis should be confirmed by the demonstration of a negative correlation between rising Hcy values and declining TTR plasma levels [25,26] in aged subjects. The liver may similarly endure toxic exposure and varying inflammatory, autoimmune, and degenerative processes associated with HHcy states, as described in viral hepatitis [62], cirrhosis [63], and nonalcoholic steatosis [64]. The replacement of normal parenchymal cells by fatty, fibrotic, or inflammatory material would lead to marked intrahepatic dysregulation of Met metabolism, as recently documented in mouse experiments showing that diet-induced steatofibrosis is associated with HHcy status and a 30% depletion of the intrahepatic Met pool size [65]. Total body REE may be reconstructed from the detailed participation of individual LBM constituents [66]. Because both musculature and liver generate 50% of the basal metabolic expenditure [43], the additional contribution of intestinal mucosa (10%) [67] and lymphocytes (7%) [68] represents two-thirds of total body REE. The intestinal tract component may be subjected to regional inflammatory processes associated with HHcy response, as shown in ulcerative colitis [69] and Crohn’s disease [70], whereas white blood cells exhibit similar reactions in lymphoblastic leukemia [71]. Comparable HHcy upsurges may result from brain damage [72], myocardial infarction [73], lung neoplasia [74], and acute pancreatitis [75]. The cutaneous tissue, which ranks second within the structural compartment, may be injured by exfoliative dermatitis [76] or open skin sores [77] while the vast network of vessels that irrigate the human body may experience local damage everywhere [78]. Taken together, these clinical morbidities cover almost all LBM tissues and account for nearly all the total body REE. Despite apparent disparity, all LBM organs possess the same enzymatic equipment to complete TS and RM processes (taking the brain [79], gastrointestinal tract [80], and white blood cells [81], as examples). We postulate that any LBM component submitted to any stress disorder (e.g., sepsis, trauma, burns, neoplasia) undergoes depletion of its N stores and Met pool size, which might endanger defense and repair systems. Methionine synthase, folate, and cobalamin are ubiquitously distributed in LBM tissues, which can therefore fulfill normal RM processes under relaxed conditions but fail to complete full RM activities during inflammatory disorders owing to the absence of BHMT in LBM tissues [14]. The biological consequences of the BHMT defect were experimentally clarified in rat models using intraperitoneal injection of a specific chemical inhibitor (*S*-(α-carboxybutyl)-dl-homocysteine) that downregulates activity of the liver methyltransferase by 90%, resulting in a 7-fold increase of circulating Hcy [82]. Moreover, a knockout mouse model with BHMT gene deletion (*Bhmt***^−/−^**) had a significant 8-fold Hcy increase in the plasma and a 6-fold Hcy increase in intrahepatic tissue [83]. This last working group concluded that BHMT has, under latent conditions, RM potential prevailing over MS capacity, which does not compensate for defective Met production when BHMT is lacking or inoperative [84]. Based on these animal experiments, we postulate that each LBM component—with the sole exception of renal tissues endowed with normal BHMT activity [14]—may only locally trigger a sluggish inflammatory response due to poor MS reactivity and blunt RM aptitude to restore intracellular Met losses. The bulk of Hcy flooding into the extracellular space is eliminated in the kidneys [85], whereas the remaining fraction is removed by hepatocytes and redirected either toward RM processes to provide additional Met sources or channeled along the TS pathway to yield Cys [86] under the regulatory control of CBS and CGL. We postulate that quiescent BHMT behavior in LBM tissues followed by the substantial release of Hcy fluxes during inflammatory disorders supports the sensing mechanism concept. Hcy operates as a biomarker stimulating liver RM capacities, providing additional production of Met molecules that are exported back to damaged tissues to meet increased in situ demands.

These data are consistent with recent findings on CBS conformation and functioning. CBS is a homotetrameric enzyme comprising 551 AA residues subdivided into three distinct domains as follows: (1) a N-terminal heme-binding domain (AA 1–70) implicated in the maintenance of cellular redox balance and enhanced ROS defense [87]; (2) a highly conserved central catalytic core (AA 71–413) containing the PLP co-factor [88]; and (3) a C-terminal regulatory region (AA 414–551) serving as a binding site for allosteric SAM activation [89]. CBS may be subjected to mutation processes, yielding allele species that undergo misfolding and changes in SAM-induced activities [90]. Two sets of SAM-binding sites have been described that are involved in kinetic stabilization and enzyme activation of the C-domain [91]. A large genome investigation has recently identified a coding gene revealing a single-nucleotide polymorphism associated with Hcy plasma levels in intron 14 of CBS [92]. A corroborative case-control study confirmed the presence in this intron 14 of a 31 base-pair (BP) variable-number tandem repeat (VNTR) that is associated with high Hcy plasma levels, conferring susceptibility to severe sepsis [93]. We assume that these last data constitute the missing link, allowing for closure of the cycle of Met→Hcy→Met fluxes in inflammatory disorders.

## 6. Conclusions

In protein-depleted states, downregulation of SAM causes CBS inhibition, hence promoting upstream sequestration of Hcy in biological fluids and favoring Hcy→Met remethylation processes by means of increased methyltransferase activities. These compensatory attempts are efficient and operate early as shown by mouse experiments indicating that liver CBS activity is downregulated by half whereas that of BHMT is nearly doubled after only 3 days of Met shortage [34]. Under protein-restricted conditions, most enzyme activities controlling Met metabolism are downgraded, with the sole exception of remethylating processes, thus emphasizing that, even in Met deprivation states, homeostasis of this *S*-containing IAA is safeguarded and benefits from high metabolic priority [4]. Dietary Met is transported to liver parenchymal cells along *short-loop* recovery pathways, using intestinal mucosa and splanchnic vasculature as relay steps.

In contrast, most TM, TS, and RM pathways are overstimulated throughout the course of inflammatory disorders [4,51]. Muscle mass and liver are the two main LBM organs, each contributing to a quarter of the total body REE [43,44], with the remaining half being subdivided into all other LBM constituents [44,66]. Taken separately, each LBM item may be subjected to a stress disorder of any cause, thereby setting in motion local defense and repair responses. This rehabilitation task is associated with a marked elevation of the Hcy biomarker [55], reflecting the duration and severity of the stressful burden [56] and operating as a signal that sounds the alarm to rescue inflamed tissues from the risk of peripheral Met depletion. In this complex metabolic scenario [88,89], the liver uses the structural and functional versatility of the CBS enzyme to maintain a balance between a multitude of distinct, divergent, and sometimes opposite impulses, in particular, when malnutrition and inflammatory influences coexist. LBM components alone are unable to control such multifunctional metabolic reactions, which require connection with central regulatory control systems exerted by the liver. As a result, hepatic parenchymal cells provide additional sources of Met molecules that are readily driven towards the site of inflamed tissues using systemic circulation and a *long-loop* circuit.

LBM downsizing, shrinking of Met pool sizes, and declining plasma TTR values occur in most clinical morbidities. Protein-depleted states and inflammatory disorders follow dualistic and unrelated physiopathological mechanisms leading, respectively, to allosteric inhibition of CBS and impairment of BHMT activities, followed by upstream accretion of Hcy in biological fluids. Considering the well-established deleterious effects of Hcy on vascular [94,95] and neural [96,97] tissues**,** HHcy states must be regarded as the dark side of these helpful RM processes. Subjects who are submitted to chronic protein deprivation or long-lasting inflammatory diseases incur increased risks to develop Hcy-induced harmful consequences.

The unique metabolic and structural roles played by Met in mammalian tissues were previously documented by Canadian workers a decade ago [98]. The present review corroborates and enlarges the scope by integrating the most recent findings described in disease states characterized by intricacy of malnutrition and inflammation components, providing a comprehensive explanation for most alterations affecting Met and Hcy metabolism.
